# Supplementary material for: Isotopic and genetic methods reveal the role of the gut microbiome in mammalian host essential amino acid metabolism
Source: Proc Biol Sci. 2020 Mar 4;287(1922):20192995. doi: 10.1098/rspb.2019.2995 (PMC7126075; doi:10.1098/rspb.2019.2995)
Supplement: Newsome et al. ESM [file rspb20192995supp1.pdf]

Electronic Supplemental Material (ESM)
Isotopic and genetic methods reveal the role of the gut microbiome in mammalian host essential amino acid metabolism

Experimental Design. Weanling mice were housed at the University of New Mexico Animal Research Facility. Experimental diets were thoroughly mixed with water into a slurry so that mice would not selectively consume particular dietary components. Mice were fed an *ad libitum* daily ration of 10g of dry food per mouse. Food and water were replaced daily, and mice were weighed weekly to monitor growth and health. The mean increase in mouse body mass (g) as a percentage of original body mass on the first day of the experiment varied among diet treatments: Diet #1 (40P:40C): +108.6%, Diet #2 (21P:45C): 57.4%, Diet #3 (12P:55C): 47.4%, Diet #4 (9P:75C): 62.7%; numbers in parentheses are approximate weight percent protein (P) and carbohydrate (C) contents of each diet treatment (Table S1, Fig. S1). However, after the weanling mice had acclimated to the experimental diets, the rate of mass gain between Week 4 and Week 15 of the experiment did not vary among diet treatments (Fig. S1).

Gut Microbiome Community Composition. Unique 16S rRNA gene sequences or operational taxonomic units (OTUs) were identified in Quantitative Insights into Microbial Ecology (QIIME, version 1.9.1) [1] by the 97% DNA identity criterion using UCLUST [2]. A representative sequence was picked from each OTU and aligned using the PyNAST aligner [3] and the Greengenes core set [4] and given taxonomic assignments using the Ribosomal Database Classifier program [5]. In addition, amplicon sequence variants (ASVs, sequences that share 100% DNA similarity), were identified using Dada2 [6]. Patterns in microbial communities based on OTUs and ASVs were assessed using nonmetric multidimensional scaling (NMDS) using Bray-Curtis distances within the Vegan library *in* R [7]. Community compositional differences among individual mice were tested using a Monte Carlo significance test performed in QIIME (beta_significance.py) determined on 100 randomizations of the unweighted unifrac distances of each pairwise sample comparison. Differences in microbial community composition among the diet treatments were analyzed within QIIME by Random Forests [8] and tested with an Analysis of Similarity (ANOSIM) run for 999 permutations also using Bray-Curtis similarity matrices. The statistical significance of explanatory variables was assessed via the Adonis test, as implemented in the Vegan R package [9].

DNA sequencing of the 16S rRNA gene resulted in 864,218 sequences after filtering of low-quality sequences and chimeras. The number of sequences ranged from 5,660 to 217,522 per sample, (mean = 43,211). These sequences represent 30,782 operational taxonomic units (OTUs) based on the 97% DNA:DNA criterion and 2,604 exact amplicon sequence variants (ASVs; [10]). To compensate for uneven coverage depth among samples, data were rarefied to 5,660 sequences per sample, which represented 8,664 OTUs and 1,804 ASVs. The results presented here are based on analysis of rarefied OTUs (97% DNA:DNA similarity) rather than ASVs (except where noted) because OTUs are a more conservative estimate of diversity. Furthermore, we currently have no ecological knowledge of this specific system that points to ASVs being a relevant population definition for these communities. However, general patterns observed in our data were similar regardless of whether we used OTUs or ASVs (Fig. S2), which is consistent with other studies where the two criteria were compared [11]. The Good's coverage statistic calculated from OTUs ranged from 0.87 to 0.95, indicating that the majority of bacterial diversity in the amplicons was detected [12]. Gut bacterial diversity increased with dietary protein content (Fig. S3) regardless if we used a richness criterion (observed OTUs) or the inverse Simpson metric.

One limitation of our experiment was that mice were co-housed, which could potentially result in the homogenization of gut microbiota communities by diet [13]. However, the diversity and community composition of the gut microbiota among the individual mice within each cage varied significantly (Fig. 2D, p-values < 0.01). For at least 80% of the pairwise comparisons of the individuals within a diet, except in Diet #1 (40P:40C) where only 50% differed significantly, indicating that any cage effect in this experiment was negligible.

Contribution of the Microbial Community Composition to Host Tissue Synthesis. The degree to which microbial community composition impacted host tissue synthesis was investigated by first performing a principle components analysis based on OTU-level Bray-Curtis dissimilarities from mice within each diet treatment. The resulting eigenvalues (site scores) were then forward selected [14] using vegan's

ordiR2step function [7]. The generated response data ($\delta^{13}C$ values of AA_{ESS}) were analyzed as a function of the eigenvalues by canonical redundancy analysis (RDA). The amount of variance explained was determined using the adjusted coefficient of multiple determination (R^2_a). R^2_a has been shown to be an unbiased estimate of the explanatory power of a set of variables [15]. The significance of results was assessed through 1000 permutations.

*Amino Acid $\delta^{13}C$ Analysis***.** Mouse muscle tissue was lipid-extracted with three ~24 hour soaks in a 2:1 chloroform:methanol solution, rinsed repeatedly in deionized water, and lyophilized. Dried lipid-extracted mouse muscle (~5mg), dietary casein (~5mg), or cornmeal (~20mg) was hydrolyzed to AAs in 1 ml of 6N hydrochloric acid at 110ºC for ~20 hours. Hydrolyzed cornmeal samples were passed through a cation exchange resin column (Dowex 50WX8 100-200 mesh) to isolate amino acids from other metabolites [16]. Hydrolyzed AAs were then derivatized to N-trifluoroacetic acid isopropyl esters [16,17] and analyzed in triplicate; a set of two internal AA isotope standards were analyzed for every three unknown samples.

Isotopic results are expressed as δ values, $\delta^{13}C = [(R(^{13}C/^{12}C)_{unknown} - R(^{13}C/^{12}C)_{standard}) / R(^{13}C/^{12}C)_{standard}]$, where $R_{unknown}$ and $R_{standard}$ are the $^{13}C/^{12}C$ ratios of the sample and standard, respectively. The internationally accepted standard is Vienna-Pee Dee Belemnite limestone (V-PDB) for carbon and the units are expressed as per mil (‰). The average within-run standard deviation of $\delta^{13}C$ values of this standard ranged from 0.3‰ (Leu and Ile) to 0.5‰ (Thr).

Using this derivatization method described above, measurements of $\delta^{13}C$ value for a given amino acid include carbon from both the amino acid and derivatization reagents (e.g., isopropanol and N-trifluoroacetic acid), which can be heavily fractionated depending on reaction mechanisms for each amino acid. Amino acid standards of known $\delta^{13}C$ composition were derivatized and analyzed along with each batch of samples. The results of the standards reflect the effect of carbon added by the derivatization reagents and provided the information required to calculate the $\delta^{13}C$ value of amino acid carbon in the samples. Individual amino acid $\delta^{13}C$ values ($\delta^{13}CAA_{sample}$) were calculated using the equation:

$$\delta^{13}CAA_{sample} = \delta^{13}CAA_{dsa} - \delta^{13}CAA_{dst} + \delta^{13}CAA_{std}\ (p_{std}) / p_{std}$$

The terms $\delta^{13}CAA_{dsa}$ and $\delta^{13}CAA_{dst}$ refer to the derivatized sample and standard respectively, and $\delta^{13}CAA_{std}$ refers to the underivatized standard. p_{std} is equal to the proportion of carbon in the derivative that was sourced from the amino acid [17,18]. We resolved twelve amino acids using these methods, six of which are non-essential for most eukaryotes: glycine (Gly), alanine (Ala), aspartic acid (Asp), glutamic Acid (Glu), proline (Pro), and tyrosine (Tyr) and six amino acids that are considered essential, including threonine (Thr) valine (Val), leucine (Leu), isoleucine (Ileu), phenylalanine (Phe), and lysine (Lys). Glutamine and Asparagine are converted to glutamic acid and aspartic acid respectively during hydrolysis and derivatization.

*Mixing Models***.** We used a series of mixing models to estimate the microbial contribution of essential amino acids used by mice in each diet treatment to build muscle tissue. First, we applied a mixing model:

$$\delta^{13}C_{Diet\ ESS\text{-}AA} = (p_{Cornmeal\ ESS\text{-}AA})\ X\ [X\ \delta^{13}C_{Cornmeal\ ESS\text{-}AA} + (p_{Casein\ ESS\text{-}AA})\ X\ \delta^{13}C_{Casein\ ESS\text{-}AA} \quad \text{(Model \#1)}$$

where $p_{Cornmeal\ ESS\text{-}AA}$ and $p_{Casein\ ESS\text{-}AA}$ is the proportion of each essential amino acid in cornmeal and casein respectively, while $\delta^{13}C_{Cornmeal\ ESS\text{-}AA}$ and $\delta^{13}C_{Casein\ ESS\text{-}AA}$ are the carbon isotope values of each AA_{ESS} in the two sources of protein (cornmeal and casein) available in each diet treatment (Table S2). The data used to calculate $p_{Cornmeal,}$ p_{Casein}, $\delta^{13}C_{Cornmeal}$, $\delta^{13}C_{Casein}$ for each amino acid is reported in Tables S2 and S3; also see Table S3 for calculated $\delta^{13}C_{Diet}$ values for each amino acid. Note that cornmeal was only 10% protein by weight. This model generated treatment-specific $\delta^{13}C$ values for amino acids that could be directly routed from diet, which for most diet treatments was overwhelmingly casein but did include a significant cornmeal derived protein component in the low (5%) protein diet treatment that contained 40% cornmeal.

We then used another mixing model to estimate the proportion of each amino acid in mouse muscle that was directly routed from dietary protein versus that sourced from carbohydrates by gut bacteria:

$$\delta^{13}C_{Muscle\ ESS\text{-}AA} = (p_{Diet\ ESS\text{-}AA}) \times \delta^{13}C_{Diet\ ESS\text{-}AA} + (p_{Bacteria\ ESS\text{-}AA}) \times \delta^{13}C_{Bacteria\ ESS\text{-}AA} \quad \text{(Model \#2)}$$

where $\delta^{13}C_{Diet\ ESS\text{-}AA}$ was estimated using Model #1 and $\delta^{13}C_{Bacteria\ ESS\text{-}AA}$ was estimated using AA-specific isotopic fractionation factors reported by Larsen *et al.* 2009 [19] to constrain the $\delta^{13}C$ of AA_{ESS} that were synthesized *de novo* from dietary carbohydrates. We used the mean (±SD) $\delta^{13}C$ value of dietary cornmeal and sucrose (-12.0±0.5‰) as the carbon isotope composition of the starting substrate; see Table S4 for fractionation factors and estimated $\delta^{13}C$ of AA_{ESS} that were synthesized from dietary carbohydrates by gut bacteria. With the equation for Model #2 we can solve for $p_{Bacteria\ ESS\text{-}AA}$ and $p_{Diet\ ESS\text{-}AA}$ assuming that the sum of their contributions is equal to one; see Table S5 for results. Within-treatment error in microbial contribution was estimated by running the model separately for each individual and then calculating standard error based on estimated microbial contributions for six individual mice per treatment.

An important caveat to our estimates of the microbiome contribution to host muscle synthesis is that we used AA-specific estimates of carbon isotope discrimination between bacterial cells and their carbon source ($\Delta^{13}C_M$, [19]) for microbial taxa (Actinobacteria) that are generally abundant in mammalian guts [20–22], but do not constitute a dominant microbial phyla in the guts of mice in our experiments (Fig. 2). Note that $\Delta^{13}C_M$ for individual AAs reported in [19] are similar in magnitude and direction as those observed by [23], who were the first to report $\Delta^{13}C_M$ for Proteobacteria grown on glucose as a sole energy source. With the exception of Thr ($\Delta^{13}C_M$ = +7‰), $\Delta^{13}C_M$ in [19] vary from -1‰ to -4‰, a range that is much smaller than the difference in $\delta^{13}C$ values between microbially-derived AAs and those sourced directly from dietary protein, which ranges from as little as ~7‰ for Thr in the low-protein treatments, but is typically 12–20‰ for the other AA_{ESS} in other treatments. As such, sensitivity analyses show that ~2‰ shifts in AA-specific $\Delta^{13}C_M$ only change our estimates of microbial contribution by ~10–15%.

Relative AA_{ESS} Supply and Demand. We used literature data to estimate AA demand as a percent of dry diet for a related strain of mice that were growing ~1g/day [19] and initially weighed 10–11g in a feeding trial lasted a total of two weeks. Starting weights for our mice were in the range of 19.3 to 27.9g, and our experiment lasted 16 weeks, so growth rates of the mice in our experiment (Fig. S1) were significantly lower than those reported in [24]; thus our calculations likely provide a maximum estimate of metabolic demand. We designed our experiments so that each individual was fed *ad libitum* an amount that met or exceeded their daily requirements for calories [25]; note that mice were caged communally by diet treatment so we must assume individual intake rates were constant among mice and across time. For the following calculations, we considered both casein and cornmeal because the latter source of protein contributed a sizable share (~44%) of the dietary protein available to mice in the low-protein diet treatment (Diet #4, Table S6).

To estimate AA supply, we used data for casein reported in [26] to calculate digestibility on an individual AA basis (range: 87 to 94%), which were used with casein [AA] data to yield an average amount (g) of each AA digested by each mouse for each diet treatment over the entire experiment. For cornmeal, we used digestibility data [26] for the protein zein, which represents 50% of the protein in corn [27]. We then used cornmeal and casein [AA] data to yield an average amount (g) of each AA digested by each mouse for each diet treatment over the entire experiment. Amounts (g) of each AA available from casein and cornmeal were summed for each diet treatment, and then converted into a % relative to the total amount of dry diet (1200g) fed to mice in each treatment over the course of the experiment. Similar supply calculations were performed for Asp.

Table S1. Relative weight percent proportions of dietary ingredients used in the four diet treatments and associated $\delta^{13}C$ data; error (SD) for $\delta^{13}C$ values are equal to analytical precision (±0.2‰).

Ingredient	Diet #1	Diet #2	Diet #3	Diet #4	$\delta^{13}C$
Casein	0.40	0.20	0.10	0.05	-26.5
Cornmeal	0.05	0.10	0.20	0.40	-12.0
Sucrose	0.36	0.36	0.36	0.36	-12.2
Cellulose	0.10	0.25	0.25	0.10	-25.6
Corn Oil	0.01	0.01	0.01	0.01	-11.8
Brewer's Yeast	0.02	0.02	0.02	0.02	-23.5
USP Fortification Salt	0.04	0.04	0.04	0.04	–
Vitamin Fortification Mixture	0.01	0.01	0.01	0.01	-12.9

Table S2. $\delta^{13}C$ values and associated weight percent concentrations of AA_{ESS} in dietary casein and cornmeal protein. Data for casein and cornmeal amino acid concentrations are from Lauer and Baker [28] and USDA [29] respectively.

Essential Amino Acid	Casein $\delta^{13}C$	Casein [AA]	Cornmeal $\delta^{13}C$	Cornmeal [AA]
Threonine	-18.0	5.2	-1.0	3.8
Valine	-31.0	8.9	-22.1	5.1
Leucine	-33.9	13.5	-24.8	12.3
Isoleucine	-29.2	7.0	-14.1	3.6
Phenylalanine	-32.8	5.9	-16.9	4.9
Lysine*	-25.3	8.7	-18.8	2.8

Table S3. Data used to calculate variables in Model #1. The concentration of protein in casein (100%) and cornmeal (10%) was used to calculate total protein content. Concentrations of each AA_{ESS} in casein and cornmeal (Table S2) were multiplied by the concentration of protein in each source to estimate the relative amount of each amino acid in casein and cornmeal. These amounts were converted into proportions of each source (p_{Casein} or $p_{Cornmeal}$) and along with with AA_{ESS} $\delta^{13}C$ values for each protein source (Table S2) used to estimate $\delta^{13}C$ values of AA_{ESS} available in each diet treatment ($\delta^{13}C_{Diet\ ESS\text{-}AA}$).

	**Diet #1 (40P/40C)**	**Diet #2 (21P/45C)**	**Diet #3 (12P/55C)**	**Diet #4 (9P/75C)**
Casein %	40.0	20.0	10.0	5.0
Cornmeal %	5.0	10.0	20.0	40.0
Cornmeal Protein %	0.5	1.0	2.0	4.0
Total Protein Content %	40.5	21.0	12.0	9.0
**Casein**				
Threonine	208.0	104.0	52.0	26.0
Valine	356.0	178.0	89.0	44.5
Leucine	540.0	270.0	135.0	67.5
Isoleucine	280.0	140.0	70.0	35.0
Phenylalanine	236.0	118.0	59.0	29.5
Lysine	348.0	174.0	87.0	43.5
**Cornmeal Protein**				
Threonine	1.9	3.8	7.6	15.2
Valine	2.6	5.1	10.2	20.4
Leucine	6.2	12.3	24.6	49.2
Isoleucine	1.8	3.6	7.2	14.4
Phenylalanine	2.5	4.9	9.8	19.6
Lysine	1.4	2.8	5.6	11.2
P(casein) Threonine	0.991	0.965	0.872	0.631
P(cornmeal) Threonine	0.009	0.035	0.128	0.369
P(casein) Valine	0.993	0.972	0.897	0.686
P(cornmeal) Valine	0.007	0.028	0.103	0.314
P(casein) Leucine	0.989	0.956	0.846	0.578
P(cornmeal) Leucine	0.011	0.044	0.154	0.422
P(casein) Isoleucine	0.994	0.975	0.907	0.709
P(cornmeal) Isoleucine	0.006	0.025	0.093	0.291
P(casein) Phenylalanine	0.990	0.960	0.858	0.601
P(cornmeal) Phenylalanine	0.010	0.040	0.142	0.399
P(casein) Lysine	0.996	0.984	0.940	0.795
P(cornmeal) Lysine	0.004	0.016	0.060	0.205
**$\delta^{13}C_{Diet\ ESS\text{-}AA}$**				
Threonine	-17.9	-17.4	-15.9	-11.7
Valine	-30.9	-30.7	-30.1	-28.2
Leucine	-33.8	-33.5	-32.5	-30.1
Isoleucine	-29.1	-28.8	-27.8	-24.8
Phenylalanine	-32.6	-32.1	-30.5	-26.4
Lysine	-25.2	-25.2	-24.9	-23.9

Table S4. Essential amino acid specific fractionation factors ($\Delta^{13}C$) from Larsen et al. [19] for Actinobacteria and *Rhodococcus* grown on a medium with sucrose as the sole carbon source. Also shown are the estimated $\delta^{13}C$ values of each essential amino acid synthesized from dietary carbohydrates (cornmeal and sucrose) that had a mean (±SD) carbon isotope value of -12.0±0.5‰. Asterisk denotes that a $\Delta^{13}C$ of 0.0‰ reported by Abelson and Hoering [23] was used for Lys.

Essential Amino Acid	$\Delta^{13}C$	$\delta^{13}C$
Threonine (Thr)	7.0	-5.0
Valine (Val)	-2.0	-14.0
Leucine (Leu)	-1.1	-13.1
Isoleucine (Ile)	-2.4	-14.4
Phenylalanine (Phe)	-4.0	-16.0
Lysine (Lys)*	0.0	-12.0

Table S5. $\delta^{13}C$ data and relative proportions (p) of diet versus microbial contributions for each amino acid; sample size for muscle data is six mice per treatment. $\delta^{13}C$ values of essential amino acids (AA_{ESS}) synthesized by bacteria from dietary carbohydrates (Bacteria AA_{ESS} $\delta^{13}C$) are also shown in Table S2. Mean (±SD) $\delta^{13}C$ values of AA_{ESS} in mouse muscle (Muscle AA_{ESS} $\delta^{13}C$) and dietary protein ($\delta^{13}C_{Diet\ ESS\text{-}AA}$) were directly measured; see Table S3 for how $\delta^{13}C_{Diet\ ESS\text{-}AA}$ were calculated. Mean (±SE) relative proportions of diet versus microbial contributions were calculated using the models described in the main text and the supplemental section *Mixing Models* (above).

	Thr	Val	Leu	Ile	Phe	Lys
Bacteria AA_{ESS} $\delta^{13}C$	-5.0	-14.0	-13.1	-14.4	-16.0	-12.0
Diet #1						
Muscle AA_{ESS} $\delta^{13}C$ (±SE)	-17.6±1.0	-24.8±1.0	-30.6±0.5	-24.9±0.4	-29.4±0.6	-24.9±0.6
$\delta^{13}C_{Diet\ ESS\text{-}AA}$	-17.9	-30.9	-33.8	-29.1	-32.6	-25.2
p_{Diet} AA_{ESS} (±SE)	97.7±8.2	63.9±5.9	84.5±2.8	71.4±2.9	80.7±3.7	97.7±4.8
$p_{Bacteria}$ AA_{ESS} (±SE)	2.3±8.2	36.1±5.9	15.5±2.8	28.6±2.9	19.3±3.7	2.3±4.8
Diet #2						
Muscle AA_{ESS} $\delta^{13}C$ (±SE)	-17.3±0.6	-24.6±1.4	-31.1±0.3	-25.0±0.5	-30.0±0.4	-24.7±0.7
$\delta^{13}C_{Diet\ ESS\text{-}AA}$	-17.4	-30.7	-33.5	-28.8	-32.1	-25.2
p_{Diet} AA_{ESS} (±SE)	99.2±5.5	63.5±8.6	88.2±2.0	73.6±4.0	87.0±3.2	96.2±5.7
$p_{Bacteria}$ AA_{ESS} (±SE)	0.8±5.5	36.5±8.6	12.0±2.0	26.4±4.0	13.0±3.2	3.8±5.7
Diet #3						
Muscle AA_{ESS} $\delta^{13}C$ (±SE)	-14.0±0.6	-24.1±1.1	-29.9±0.4	-23.8±0.4	-27.4±0.3	-23.4±0.3
$\delta^{13}C_{Diet\ ESS\text{-}AA}$	-15.9	-30.1	-32.5	-27.8	-30.5	-24.9
p_{Diet} AA_{ESS} (±SE)	82.6±5.7	62.7±6.7	86.6±2.7	70.1±3.2	78.6±2.6	88.4±3.2
$p_{Bacteria}$ AA_{ESS} (±SE)	17.4±5.7	37.3±6.7	13.4±2.7	29.9±3.2	21.4±2.6	11.6±3.2
Diet #4						
Muscle AA_{ESS} $\delta^{13}C$ (±SE)	-10.6±1.2	-20.0±0.9	-26.6±0.4	-19.7±0.7	-25.4±0.5	-22.4±0.8
$\delta^{13}C_{Diet\ ESS\text{-}AA}$	-11.7	-28.2	-30.1	-24.8	-26.4	-23.9
p_{Diet} AA_{ESS} (±SE)	83.6±18.0	42.3±6.8	79.4±2.8	51.0±6.6	90.4±5.7	87.2±6.9
$p_{Bacteria}$ AA_{ESS} (±SE)	16.4±18.0	57.7±6.8	20.6±2.8	49.0±6.6	9.6±5.7	12.8±6.9

Table S6. $\delta^{13}C$ values of non-essential amino acids in casein, cornmeal, and mice muscle from all four diet treatments; data for mice muscle are mean (±SD) $\delta^{13}C$ values from six mice from each diet treatment. Error (SD) for $\delta^{13}C$ values of casein and cornmeal protein are equal to analytical precision, which varies from ±0.3‰ to ±0.5‰ for the AA measured here.

	Gly	Ala	Asp	Glu	Pro	Tyr
$\delta^{13}C_{Casein}$	-20.1	-24.4	-25.4	-23.2	-23.9	-30.4
$\delta^{13}C_{Cornmeal}$	-15.4	-10.9	-14.0	-19.1	-14.7	-9.9
Muscle $\delta^{13}C$ (Diet #1)	-17.2±2.6	-11.8±1.1	-16.8±2.1	-15.4±2.1	-19.7±1.1	-27.8±2.7
Muscle $\delta^{13}C$ (Diet #2)	-16.6±1.6	-9.6±1.3	-15.1±2.1	-12.7±2.3	-20.0±1.8	-27.8±1.4
Muscle $\delta^{13}C$ (Diet #3)	-15.0±1.8	-8.5±1.1	-12.9±2.4	-10.8±2.7	-17.7±0.8	-26.9±2.0
Muscle $\delta^{13}C$ (Diet #4)	-14.3±1.7	-6.4±1.3	-11.1±2.4	-10.3±2.6	-15.0±1.5	-25.2±2.8

Table S7. The percentage of casein and cornmeal protein in each diet treatment used to estimate the overall amount (g) of these two sources of protein fed to mice over the course of the 4-month experiment assuming a daily *ad libitum* ration of 10g of dry food per mouse.

	Casein Protein (%)	Cornmeal Protein (%)	Casein (g)	Cornmeal Protein (g)
Diet #1	40.0	0.5	480.2	5.8
Diet #2	20.0	1.0	239.9	12.1
Diet #3	10.0	2.0	120.0	24.0
Diet #4	5.0	4.0	59.9	48.1

Table S8. Data used to calculate supply and demand of AA_{ESS} for mice in each diet treatment. See Table S2 for dietary casein and cornmeal [AA_{ESS}]. Amino acid specific digestibility data for casein and cornmeal protein are from Keith and Bell [26]; data for Lysine in cornmeal was not reported in Keith and Bell [26], so we assumed a digestibility of 72%, which is the mean digestibility of the other AA_{ESS} in this protein source. Asterisk denotes AA_{ESS} demand data reported in John and Bell [25] for a rapidly growing related strain of mice.

	Thr	Val	Leu	Ile	Phe	Lys
Casein Digestibility (%)	87	91	94	90	90	94
Cornmeal Protein Digestibility (%)	58	71	80	74	75	72
Diet #1 Total (g)	21.9	39.1	61.5	30.4	25.7	39.4
Diet #2 Total (g)	11.1	19.9	31.6	15.4	13.2	19.9
Diet #3 Total (g)	6.0	10.6	17.6	8.2	7.3	10.3
Diet #4 Total (g)	3.8	6.6	12.3	5.1	4.9	5.9
% of Dry Diet (Diet #1)	1.8	3.3	5.1	2.5	2.1	3.3
% of Dry Diet (Diet #2)	0.9	1.7	2.6	1.3	1.1	1.7
% of Dry Diet (Diet #3)	0.5	0.9	1.5	0.7	0.6	0.9
% of Dry Diet (Diet #4)	0.3	0.5	1.0	0.4	0.4	0.5
% of Dry Diet (Demand)*	0.4	0.5	0.7	0.4	0.4	0.4

Figure S1. Weekly mean weights (g) of mice by diet treatment; error bars represent standard error and sample size is six mice per treatment. All treatments gained weight during the course of the experiment, however, the degree of weight gain at the end of the experiment (Week 15) as a percentage of initial weight (Week 1) varied from 42% in Diet #3, 64% in Diet 4, 69% in Diet #2, and 103% in Diet #1. The rate of weight gain after Week 4 of the experiment, however, did not differ among diet treatments (P>0.05); observed differences in rates of weight gain prior to Week 4 likely reflect diet acclimation for the young weanling mice used in our experiment.

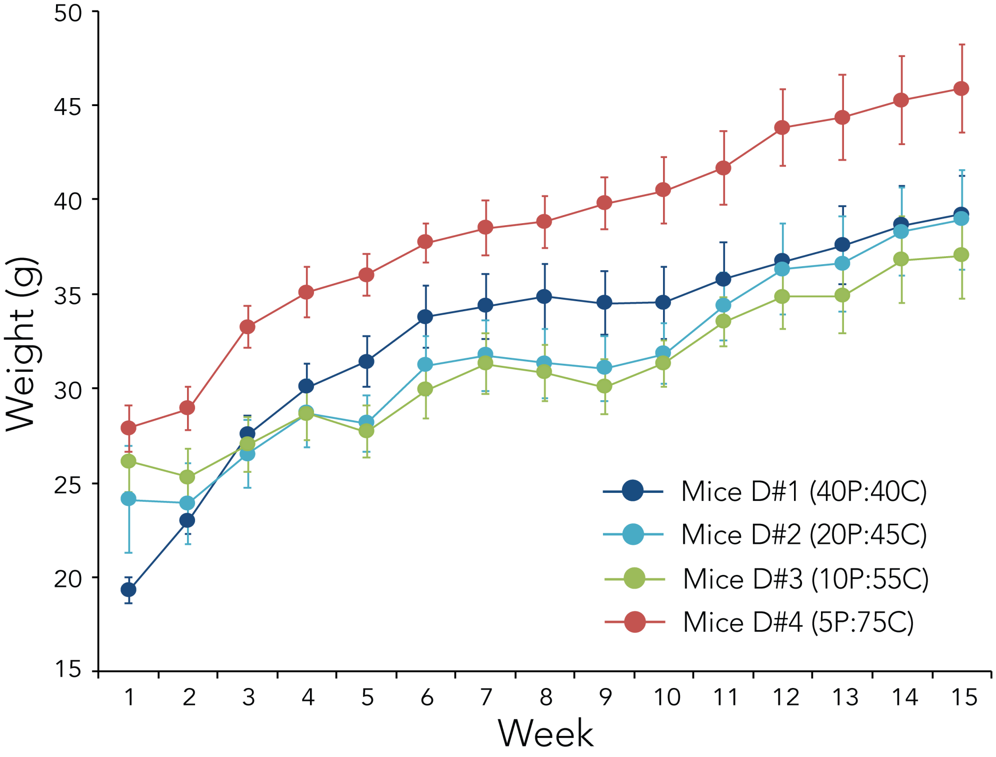

Figure S2. Non-metric multidimensional scaling (NMDS) plots of the microbial composition of ceca in mice fed one of four diets that varied in weight percent protein (P) and carbohydrate (C) content based on OTUs (97% DNA:DNA similarity, Panels A and B) and exact amplicon sequence variants (ASVs, Panels C and D) on the entire sequence dataset (Panels A and C) and rarefied to 5,660 sequences (Panels B and D). Adonis test statistic indicating the strength and significance of the relationship between the clustering observed and diet is given in each panel. Ellipses represent 95% confidence level.

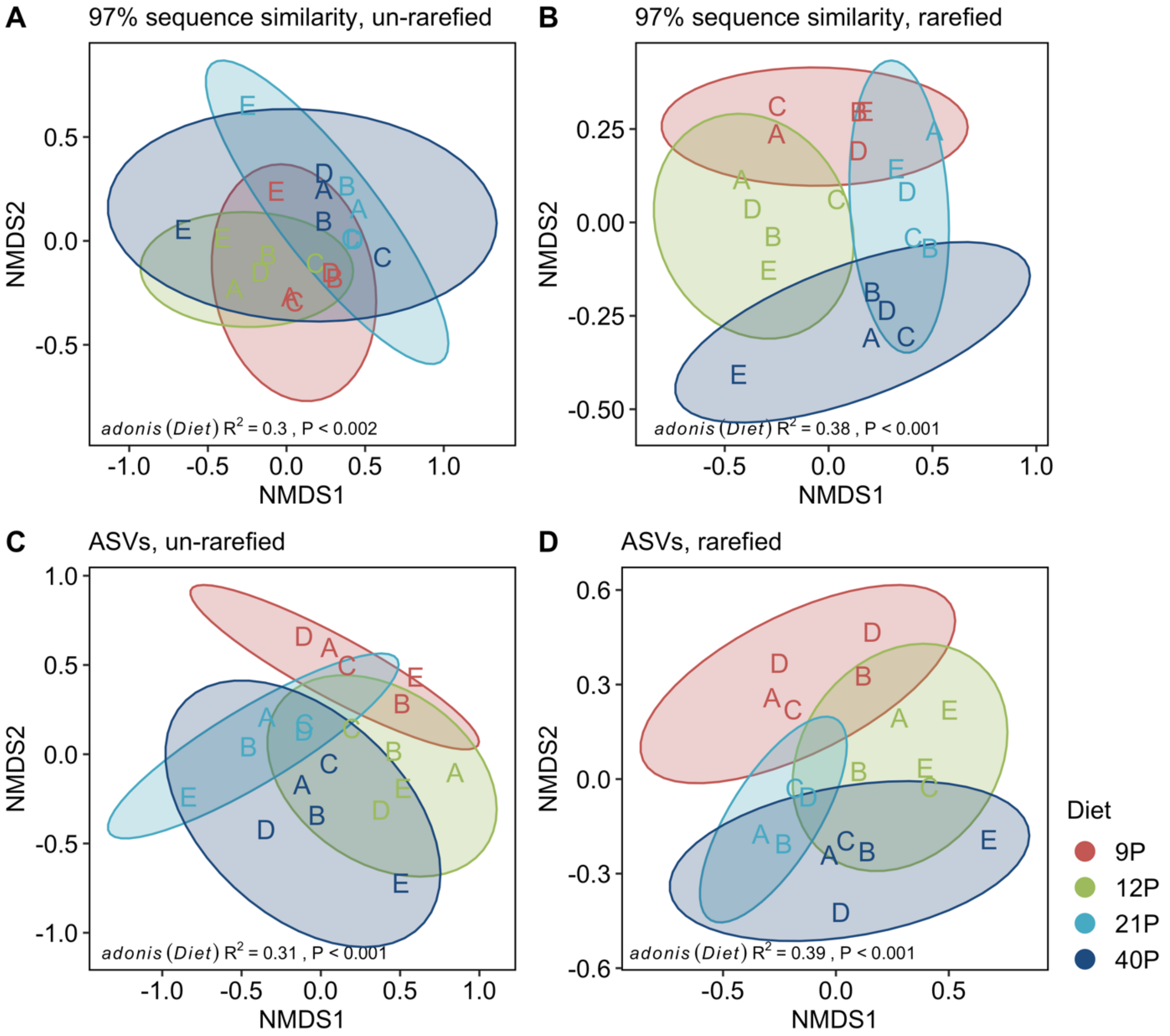

Figure S3. Box and whisker plots illustrating alpha diversity metrics by diet. Left panel indicates microbial richness (number of observed OTUs) and right panel is the inverse Simpson metric.

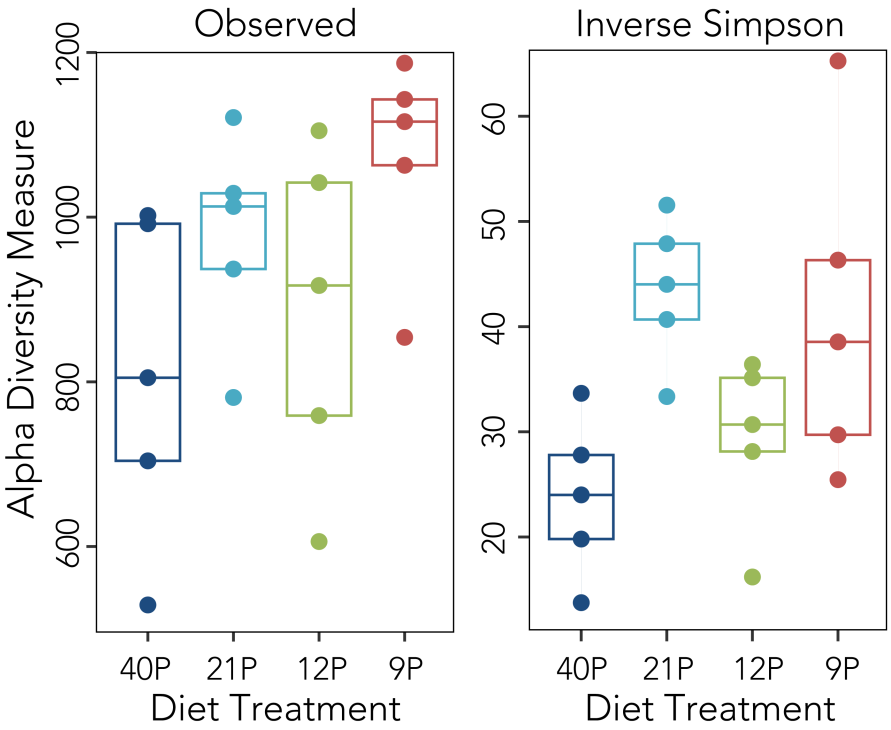

Supporting References

1. Caporaso JG, et al. (2010a) QIIME allows analysis of high-throughput community sequencing data. *Nature Methods* 7(5):335–336.
2. Edgar RC (2010) Search and clustering orders of magnitude faster than BLAST. *Bioinform* 26:2460–2461.
3. Caporaso JG, et al. (2010b) PyNAST: a flexible tool for aligning sequences to a template alignment. *Bioinform* 26:266–267.
4. DeSantis TZ, et al. (2006) Greengenes, a chimera-checked 16S rRNA gene database and workbench compatible with ARB. *Appl Environ Microbiol* 72:5069–5072.
5. Wang Q, Garrity GM, Tiedje JM, Cole JR (2007) Naïve Bayesian classifier for rapid assignment of rRNA sequences into the new bacterial taxonomy. *Appl Environ Microbiol* 73:5261–5267.
6. Callahan BJ, McMurdie PJ, Rosen MJ, Han AW, Johnson AJ, Holmes SP. DADA2: High-resolution sample inference from Illumina amplicon data. *Nat Methods*. 2016 13:581–583.
7. Oksanen J, et al. (2016) Vegan: community ecology package. *R Package Version 2.3-5* http://CRAN.R-project.org/package=vegan
8. Breiman L (2001) Random forests. *Machine Learning* 45(1):5–32.
9. Oksanen J, et al. (2010) Multivariate analysis of ecological communities in R: vegan tutorial. R package Version 1.7.
10. Callahan BJ, McMurdie PJ, Rosen MJ, Han AW, Johnson AJ, Holmes SP. (2016) DADA2: High-resolution sample inference from Illumina amplicon data. *Nat Methods* 13:581–583.
11. Glassman SI, Martiny JBH (2018) Broadscale ecological patterns are robust to use of exact sequence variants versus operational taxonomic units. *mSphere* 3: e00148-18.
12. Good IJ (1953) The population frequencies of species and the estimation of population parameters. *Biometrika* 40(3-4):237–264.
13. Deloris AA, Orcutt RP, Henry JC, Baker J, Bissahoyo AC, Threadgill DW (2006) Quantitative PCR assays for mouse enteric flora reveal strain-dependent differences in composition that are influenced by the microenvironment. *Mammalian Genome* 17: 1093-1104.
14. Blanchet FG, Legendre P, Borcard D (2008) Forward selection of explanatory variables. *Ecology* 89: 2623–2632.
15. Ohtani K (2000) Bootstrapping R 2 and adjusted R 2 in regression analysis. *Econ Model* 17: 473–483.
16. Amelung W, Zhang X (2001) Determination of amino acid enantiomers in soils. *Soil Biol Biochem* 33:553–562.
17. O'Brien DM, Fogel ML, Boggs CL. 2002 Renewable and nonrenewable resources: amino acid turnover and allocation to reproduction in Lepidoptera. *Proc Natl Acad Sci USA* 99:4413–4418.
18. Newsome SD, Fogel ML, Kelly L, Martinez del Rio CM. 2011 Contributions of direct incorporation from diet and microbial amino acids to protein synthesis in Nile tilapia. *Functional Ecology* 25(5):1051–1062.
19. Larsen T, Taylor D, Leigh M, O'Brien D. 2009 Stable isotope fingerprinting: a novel method for identifying plant, fungal, or bacterial origins of amino acids. *Ecol* 90(12):3526–3535.
20. White BA, Lamed R, Bayer EA, Flint HJ (2014) Biomass utilization by gut microbiomes. *Ann Rev Microbiol* 68:279–296.
21. Qin J, et al. (2010) A human gut microbial gene catalogue established by metagenomic sequencing. *Nature* 464:59–65.
22. D'Argenio V, Salvatore F (2015) The role of the gut microbiome in the healthy adult status. *Clin Chim Acta* 451:97–102.
23. Abelson PH, Hoering TC (1961) Carbon isotope fractionation in formation of amino acids by photosynthetic organisms. *Proc Natl Acad Sci USA* 47(5):623–632.
24. John AM, Bell JM. 1976 Amino acid requirements of the growing mouse. *J Nutr* 106:1361–1367.
25. National Research Council (1995) Nutritional requirements for laboratory animals. Fourth Edition. The National Academies Press. Washington, DC.
26. Keith MO, Bell JM. 1988 Digestibility of nitrogen and amino acids in selected protein sources fed to mice. *J Nutr* 118:561–568.

27. Shukla R, Cheryan M (2001) Zein: the industrial protein from corn. *Ind Crops Prod* 13:171–192.
28. Lauer BH, Baker BE. 1977 Amino acid composition of casein isolated from the milks of different species. *Canadian Journal of Zoology* 55:231–236.
29. United States Department of Agriculture, Agricultural Research Service. 2008. USDA National Nutrient Database for Standard Reference, Release 21. Beltsville, MD, USA.
